# Remarkably enhanced thermal transport based on a flexible horizontally-aligned carbon nanotube array film

**DOI:** 10.1038/srep21014

**Published:** 2016-02-16

**Authors:** Lin Qiu, Xiaotian Wang, Guoping Su, Dawei Tang, Xinghua Zheng, Jie Zhu, Zhiguo Wang, Pamela M. Norris, Philip D. Bradford, Yuntian Zhu

**Affiliations:** 1 Institute of Engineering Thermophysics, Chinese Academy of Sciences, Beijing, 100190, People’s Republic of China; 2 Department of Mechanical and Aerospace Engineering, University of Virginia, Charlottesville, VA 22904-4746, USA; 3 School of Materials Science and Engineering, Nanyang Technological University, 50 Nanyang Avenue, Singapore 639798, Singapore; 4 Shenhua Guohua (Beijing) Electric Power Research Institute Co., Ltd., Beijing 100025, People’s Republic of China; 5 Department of Mechanical Engineering, University of California, Riverside, CA 92521, USA; 6 China National Electric Engineering Co., Ltd., Beijing 100048, People’s Republic of China; 7 Department of Materials Science and Engineering, North Carolina State University, Raleigh, NC 27695, USA; 8 School of Materials Science and Engineering, Nanjing University of Science and Technology, Nanjing 210094, People’s Republic of China

## Abstract

It has been more than a decade since the thermal conductivity of vertically aligned carbon nanotube (VACNT) arrays was reported possible to exceed that of the best thermal greases or phase change materials by an order of magnitude. Despite tremendous prospects as a thermal interface material (TIM), results were discouraging for practical applications. The primary reason is the large thermal contact resistance between the CNT tips and the heat sink. Here we report a simultaneous sevenfold increase in in-plane thermal conductivity and a fourfold reduction in the thermal contact resistance at the flexible CNT-SiO_2_ coated heat sink interface by coupling the CNTs with orderly physical overlapping along the horizontal direction through an engineering approach (shear pressing). The removal of empty space rapidly increases the density of transport channels, and the replacement of the fine CNT tips with their cylindrical surface insures intimate contact at CNT-SiO_2_ interface. Our results suggest horizontally aligned CNT arrays exhibit remarkably enhanced in-plane thermal conductivity and reduced out-of-plane thermal conductivity and thermal contact resistance. This novel structure makes CNT film promising for applications in chip-level heat dissipation. Besides TIM, it also provides for a solution to anisotropic heat spreader which is significant for eliminating hot spots.

Vertically aligned carbon nanotube (VACNT) arrays potentially can be a promising thermal interface material (TIM) as their thermal conductivity along the direction of nanotube growth has been reported to be one order of magnitude higher than that of the best thermal greases or phase change materials^1^,^2^,^3^,^4^. Combined with their experimentally proven resilient mechanical performance^5^,^6^, VACNT arrays offer a unique candidate for thermal management. To accelerate the practical application of CNTs as TIMs, most efforts have been focused on developing VACNT arrays with further enhanced thermal conductivity. However, although their longitudinal thermal conductivity rivals ~80 W/mK at room temperature, most works reported the large thermal contact resistance between the tips of the VACNT arrays and the substrate as a major challenge to the application of these materials. The order of magnitude of the thermal contact resistance has been experimentally found to reach values as high as 10^−5^ K/m^2^W, caused in large part by the low surface coverage of CNTs on the heat sink^3^,^4^, thus dominating the total thermal resistance of VACNT arrays. This large thermal resistance value is comparable to the magnitude of thermal boundary resistance (TBR) existing between some solid-solid interfaces without the presence of any TIM^7^,^8^, diminishing the advantage of VACNT arrays as a TIM.

For perfectly aligned VACNT array-based systems, heat transport is one-dimensional and heat dissipation will not occur in plane^3^,^4^. In series with the volumetric thermal resistance of the CNTs, the thermal contact resistance between the VACNT tips and the heat sink likely dominates the total thermal resistance and thus significantly impedes the flow of heat to the heat sink. The primary reason for the low thermal contact resistance is the inevitably different lengths of as-grown VACNTs. This structural feature is the main culprit for the degradation of the performance in VACNT array TIM applications. The thermal contact resistance at the VACNT-heat sink interface can be reduced depending on the roughness of the top surface. One of the co-authors^6^,^9^ and others^10^ have shown that exceedingly small top surface roughness values and good tensile strength can be obtained if the VACNT arrays are transformed into flexible horizontally aligned carbon nanotube (HACNT) array films. And the produced flexible HACNT array film was shown to be stable in air for many months.

In this article we report that compact horizontal packing and transverse physical overlapping dramatically improves in-plane heat transport in HACNT arrays. The ordered alignment of CNTs and significant reduction of roughness at the top surface lead to a remarkably high thermal conductivity, 127 W/mK, along the principle CNT length direction and a reduced thermal contact resistance at the CNT array film-SiO_2_ interface, 0.8 m^2^K/MW. Meanwhile, the out-of-plane thermal conductivity is dramatically inhibited to around 4 W/mK, which fails to bring its other advantages, i.e., high in-plane transport and low thermal contact resistance, to realize performance improvement aimed for TIM. However, this unique anisotropy in thermal transport for HACNT provide a solution to another key heat issue in die-level packaging, i.e., extremely high temperature immediately below the heat source or called “hot spots” owing to high thickness-direction thermal conductivity of conventional aluminum and copper heat spreaders attached to the lower case of a microprocessor chip. The HACNT exhibiting high thermal conductivity in the plane direction (127 W/mK) and much lower thermal conductivity through the thickness direction (4 W/mK), allows it to function as both an insulator and heat spreader with the advantage to eliminate hot spots and thus protect other electronic components in microprocessor chips. This novel finding makes CNT film very promising for application in chip-level heat dissipation system. In addition, VACNT could be used as TIM, its variant HACNT can be used as micro/nanoscale anisotropic heat spreader, which is significant for retarding thermal fatigue, slimming down design and reducing weights.

## Fabrication Schemes for Flexible CNT Array Films

**Figure 1:**
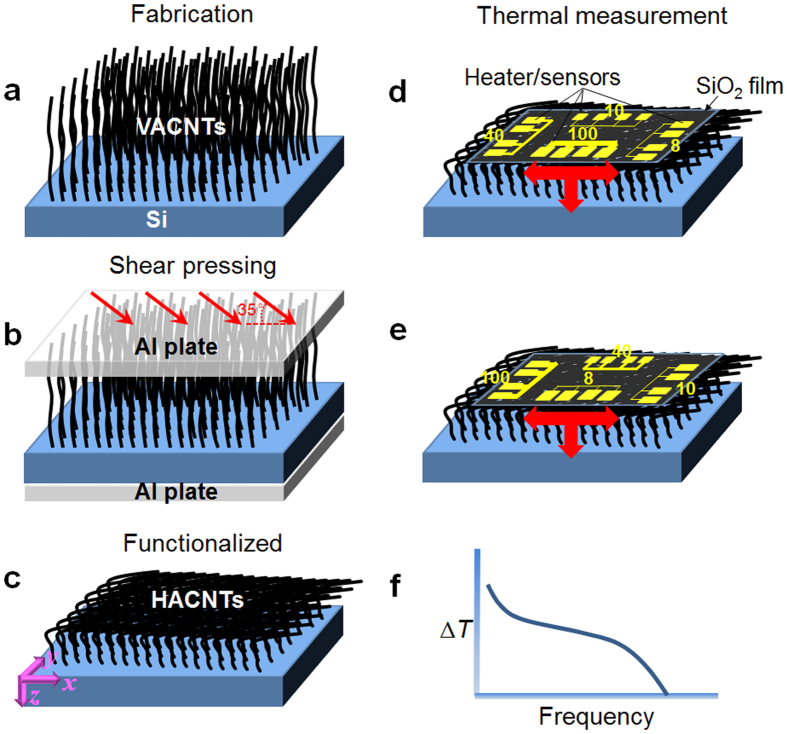
Schematics of sample preparation and thermal transport measurement. (**a**) A vertically aligned CNT array on a silicon substrate. (**b**) Shear pressing of the CNT array. (**c**) Horizontally aligned CNT arrays formed after shear pressing. (**d**,**e**) Thermal transport measurements by heating the 3 *ω* heater/sensor group deposited on HACNT arrays. The group consists of four Au micro-sensors with different widths: 100 μm, 10 μm, 40 μm and 8 μm, which were marked as *x*-100, *x*-10, *y*-40 and *y*-8 sensor for the top-right graph and *x*-40, *x*-8, *y*-100 and *y*-10 sensor for the middle-right graph as a control to check the result. (**f**) Typical measurement analysis based on temperature rise magnitude of micro-sensor in frequency domain.

The flexible horizontally aligned CNT array films were fabricated by a similar mechanical method with our previous work^6^,^9^, as shown by Fig. 1. In a typical experiment, we grew VACNT arrays on Si substrates using catalyzed chemical vapor deposition. The CNTs are approximately 1.2 mm tall and typically have 2–4 walls with average outer diameter of approximately 8.2 nm^6^,^9^. The VACNT arrays were placed on a custom pressing device with two parallel Al plates. One is fixed and the other is moveable with adjustable angle. The as-grown VACNT array was shear pressed into a HACNT array at an optimized angle of 35°^9^ in relation to the Si plane. It is to be noted that the lower pressing angle was desired to increase the horizontal shear imparted to the CNT array. However, it was found that angles lower than 35° formed non-uniform wavy CNT caused by partial separation and over shearing of CNT from the Si substrate. Thus, 35° is believed the optimal pressing angle to transfer VACNT to HACNT in our case. The pressing time was controlled to be approximately two seconds. Slower or faster pressing process was found to have no noticeable effects on the pressed film. The processed CNT array turned out to be a combination of two parts: a ~45 μm thick horizontally aligned CNT layer on the top (see Figure S1 in Supporting Information) and a vertically aligned CNT segment subject to bending buckling on the bottom^9^. The top surface of the HACNT array was then carefully polished to have acceptable surface evenness and roughness to ensure success in deposition of a 200 nm thick metal sensor for thermal characterization. This approach involved only mechanical processing of the CNT arrays, avoiding additional change in defects or impurity concentration which might accompany heat treatment^11^ or chemical processing^12^.

## Microscopic Structure Characterization and Modeling

**Figure 2:**
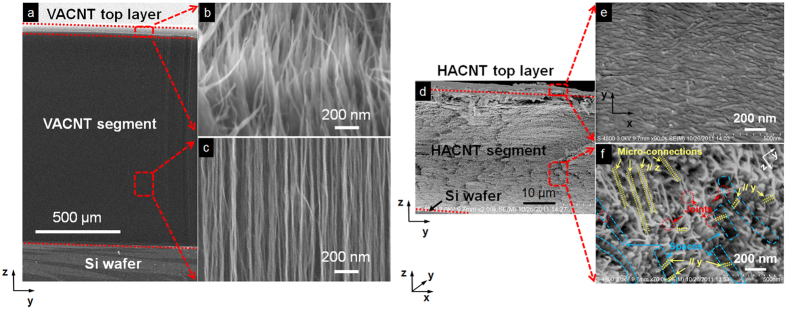
SEM images showing formed coupling structure and increased density of HACNT arrays. (**a**) FESEM image for cross-section view for VACNT and high magnification of (**b**) top layer and (**c**) VACNT segment. (**d**) FESEM image for cross-section view for HACNT and high magnification of (**e**) top layer and (**f**) HACNT segment.

The microscopic structures of both the flexible HACNT array film and its original as-grown VACNT array were characterized by field emission scanning electron microscope (FESEM) testing. Consistent with the arrays before and after shear pressing, FESEM images for cross-section view show remarkable contrast (Fig. 2a,d). High magnification SEM images for the top surface morphology and actual inner structure of the synthesized VACNT and HACNT arrays are shown in Fig. 2b,c,e,f. It can be seen that the CNTs still maintain orderly packing along the in-plane direction to a large extent and the top surface is flattened and densified (Fig. 2h,i), which is beneficial for enhancing thermal conductivity and reducing thermal contact resistance. The inner structure of the flexible HACNT array film is very complicated and could be considered a combination of a large quantity of “CNTs zones” and “spaces zones”. “Spaces zones”, which exist between “CNTs zones”, typically have a strip shape inclining along the *z* direction. “Spaces zones” could block the thermal transport along the *y* direction. “CNT zones”, which consist of numerous micro-connections and joints that are favorable to thermal transport in the *y*-*z* plane (Fig. 2f), play a vital role in energy transport. In the *z*-direction, micro-misconnections are generally made through upper-layer CNTs falling down during the inclined shear pressing process. The *y*-direction micro-connections, which can also be generated in the “CNTs zones” due to the horizontal force generated during the inclined shear pressing process, tend to form bridges across parallel CNTs. This unintended effect of *z* and *y*-direction micro-connections, therefore, brings about large transverse energy transport ability in contrast to the zero transverse transport in VACNTs^4^. At the bottom of the HACNT array, experimental observations from our work^9^ and others^13^,^14^ show that CNTs are subjected to bending buckling owing to the strain force experienced during the shear pressing, and the formed localized buckling kinks with bending angle Θ ≈ 90° actually play a major role in ensuring the structural integrity of the CNT films^15^. However, these highly distorted buckling kinks, together with flattened cross-sections of CNTs, also greatly influence both the mechanical and thermal properties around them^14^,^15^.

From the perspective of heat transport, the heat flow modes are discrete in three dimensions, and the comparison between before and after shear pressing is illustrated in Fig. 2a,d. The microscopic structure of the HACNTs is modelled as orderly overlapped 4 walled CNTs with partial inter-tube contacts along the length, as shown in Fig. 2f. Since no covalent bonds were modified in the CNTs, interfacial Kapitza resistances^16^,^17^,^18^ can be ignored and due to the very similar phonon spectra of the CNTs, the inter-tube interaction is totally attributed to weak van der Waals forces. This simple intuitive picture of CNT alignment implies that the parallel MWCNTs orderly overlap with adjacent ones at a van der Waals interaction distance (3.4 Å at equilibrium state) along the *x* axis, and thus the packing density of the HACNT arrays (or the number of transport channels) is swiftly increased by squeezing out the air originally trapped between the CNTs at the point of overlapping. The cross section in the *y*-*z* plane of the HACNT arrays is illustrated in Fig. 2d. The packing density is estimated to reach 10.1% based on 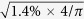, a nearly sevenfold increase as compared with the original 1.4% in the VACNT array (obtained through the comparison of the measured density of a CNT array^6^ 0.017 g/cm^3^ to that of an individual CNT 1.25 g/cm^3^, see the Methods Section for the detailed calculation process). The “spaces zones” between “CNT zones” in the *y*-*z* plane (Fig. 2f) are considered as the main obstacle to further increases in packing density for increased thermal transport channels parallel to the *x* direction.

Along the *y* axis, experimental observations revealed that parallel CNTs inevitably partly contacted with adjacent ones either by forming joints or micro-connections after shear pressing as pointed out above (Fig. 2f), which created numerous contacts enabling heat transfer. This is totally different from the situation in VACNT arrays which display no heat transfer between separated CNTs^4^ as no interactions can help to convey vibrational modes crossing the large gap between adjacent CNTs, and thus the emerging in-plane thermal transport is a unique feature for HACNTs. With the aid of joints and micro-connections, which greatly shorten the space between CNTs along the transverse direction, heat can transport between adjacent CNTs at the van der Waals contact distance. The interfaces formed between overlapped CNTs, therefore, perform like thermal boundaries, which greatly enhance phonon scattering. This structural characteristic suggests that thermal transport along the *y* direction will be much smaller than that along the *x* direction where phonons can transport directly along the CNT shells with relatively long mean free paths.

Along the *z* axis, heat transports through conduction in densely packed CNT layers from the upper “hot” CNTs to the adjacent “cold” ones. Similar to transport in the *y* axis, the *z*-direction thermal transport mainly relies on the weak van der Waals interactions between inter-tube wrapped graphene layers together with micro-connections and joints along the *z* direction. However, it is to be noted that the buckling kinks at the array bottom also hamper ballistic phonon transport along the buckled nanotubes. Computational simulation results^19^,^20^ indicate that this strain would greatly modify the lattice thermal transport parameters, i.e. reducing group velocities *υ*_*m*_ under the tension strain, or reducing phonon mean free path (mfp) *l* by inducing mismatch of the phonon modes between the flattened region and other segments under the compression strain. Therefore, it is believed that the buckling kinks at the bottom serve as a series of “thermal resistors” for heat conduction along the *z* direction (Fig. 2f). Owing to the existence of buckling kinks, *z*-direction thermal transport would be further reduced compared to the *y* direction and the quantitative effect will be discussed later.

## Thermal Transport Measurements

A modified version of 3 *ω* technique was used for this part. The experimental configuration was suggested by the anisotropic solid 3 *ω* studies of Borca-Tasciuc *et al.*^21^. Typically, a 10/95 nm thick Ti/SiO_2_ adhesion/insulation film was firstly made on the top of the HACNT arrays using magnetron sputtering. Then a 200 nm thick Au film was sputtered and patterned into a certain layout using a photolithography technique. By heating the arrays with Joule heat released by electrical current-driven micro-sensors and then monitoring the micro-sensors’ temperature response, the anisotropic thermal conductivity, thermal diffusivity of the flexible HACNT array film, thermal contact resistance between the upper SiO_2_ insulation and the HACNT array and thermal contact resistance between the HACNT array and the silicon substrate can be deduced based on a rigorous heat transfer model (see “Thermal modelling” in the Methods Section). Differential measurements were made to compare the temperature rise curves for micro-sensors with large and small widths, as shown schematically in Fig. 1d,e.

**Figure 3:**
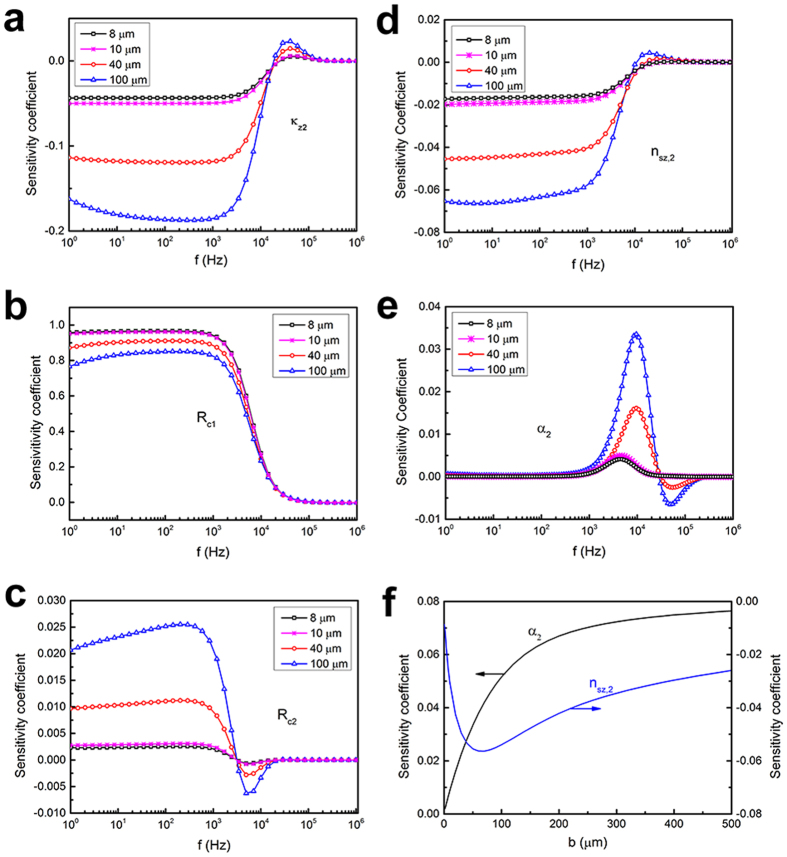
Sensitivity coefficients for thermophysical parameters of interest. Matrix of sensitivity coefficients is defined as 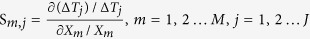, where *X*_*m*_ stands for the group of the fitted thermal properties. *M* and *J* are the total number of the fitted parameters and measured frequencies. (**a**) Out-of-plane thermal conductivity of HACNT array (*κ*_*z*2_). (**b**) TBR at the SiO_2_ film-HACNT array contact interface (*R*_*c*1_). (**c**) TBR at the HACNT array-silicon contact interface (*R*_*c*2_). (**d**) Thermal conductivity anisotropic ratio (*n*_*sz*2_). (**e**) Cross-plane thermal diffusivity (*α*_2_). (**f**) *n*_*sz*2_ and *α*_2_ with variations in half width of the 3 *ω* sensor.

A multi-parameter fitting method is often used for simultaneous extraction of several unknown parameters of interest^4^,^22^. However, our sensitivity analysis results reveal that the multi-parameter fitting scheme does not work for the present experimental configuration. The evolution for the sensitivity coefficients of four parameters: the cross-plane thermal conductivity of the HACNT array (*κ*_*z*2_), the HACNT-SiO_2_ thermal contact resistance (*R*_*c*1_), the HACNT-Si thermal contact resistance (*R*_*c*2_), and the ratio of the in-plane to cross-plane thermal conductivity (*n*_*sz*2_), shows that the sensitivity coefficients are more or less similar and the existing distinctions in the high frequency region are very small, as shown in Fig. 3a–d. In addition, as some microelectronic components in the present electrical circuit do not work properly at such high frequencies, experimental signals at frequencies above 10^4^ Hz become unreliable. Insufficient experimental data further adds to the difficulty in successful discrimination of all four parameters, *κ*_*z*2_, *R*_*c*1_, *R*_*c*2_ and *n*_*sz*2_, through simultaneous fittings. Given the sensitivity coefficient of the thermal diffusivity of the HACNT array (*α*_2_) presents a fully different dependence on frequency, only two parameters can be simultaneously extracted for the present experimental conditions, one is *α*_2_ and the other can be anyone of the remaining four parameters (*κ*_*z*2_, *R*_*c*1_, *R*_*c*2_, *n*_*sz*2_). We investigated feasible deduction methods to extract multiple parameters using a 3 *ω* method at the present experimental condition. Multi-sensor-width 3 *ω* measurements were critical in determining, independently, three-dimensional thermal properties and thermal contact resistances for the HACNT array and its interfaces.

**Figure 4:**
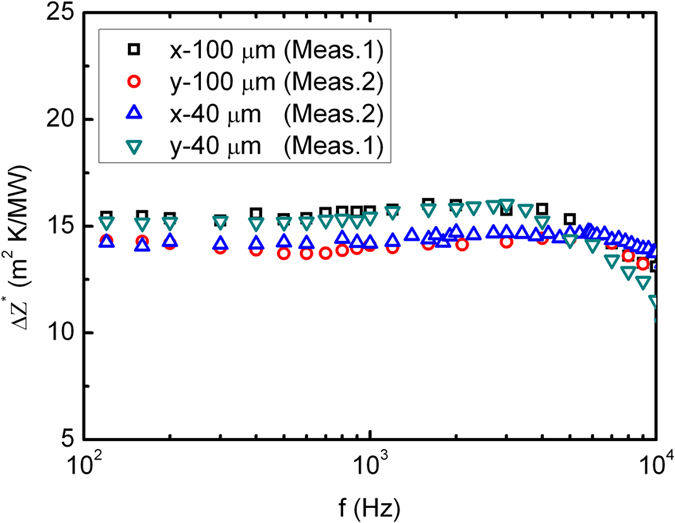
Experimental thermal impedance data at multiple frequencies. Deduced thermal impedance magnitudes according to observed 3 *ω* voltage harmonics (the first and the third harmonics) for the *x*-100, *y*-100, *x*-40 and *y*-40 sensor.

To determine the anisotropic thermal properties independently, we made 3 *ω* measurements based on differential width micro-sensors at modulation frequencies between 0.03 and 30 kHz. Increasing the sensor width increases the sensitivity for distinguishing the out-of-plane thermal conductivity of the HACNT arrays *κ*_*z*2_ and overall thermal contact resistances for the HACNT array and its interfaces (*R*_*c*1_ + *R*_*c*2_) according to our sensitivity analysis in Fig. 3a–c. Experimental results for the 100 and 40 μm wide sensors, denoted *x*-100, *y*-100, *x*-40 and *y*-40 sensor, are shown in Fig. 4. The differential thermal impedance magnitudes between two measured samples were due to the different sample thickness verified by FESEM (40 μm for sample 1 and 45 μm for sample 2). Results from both 100 and 40 μm wide sensors have very close thermal impedance, and thus can be used for determining thermal conductivity and thermal contact resistance. We also see that the differential thermal impedance is only weakly dependent on modulation frequency. To analyze these results we referred to the 2D film-on-substrate heat conduction model given by Majumdar *et al.*^23^. The out-of-plane thermal conductivity for the HACNT array *κ*_*z*2_ is determined to be 4.0 W/mK, indicating extremely low heat transfer between completely overlapped CNT layers along the *z* axis after shear pressing. In addition, the overall thermal contact resistances for the HACNT array and its interfaces (*R*_*c*1_ + *R*_*c*2_) are estimated to be 4.3 m^2^K/MW, much smaller than the VACNT counterpart (~16 m^2^K/MW)^4^, indicating a strong enhancement in interface thermal transport owing to the densified and flattened surface of the HACNT.

**Figure 5:**
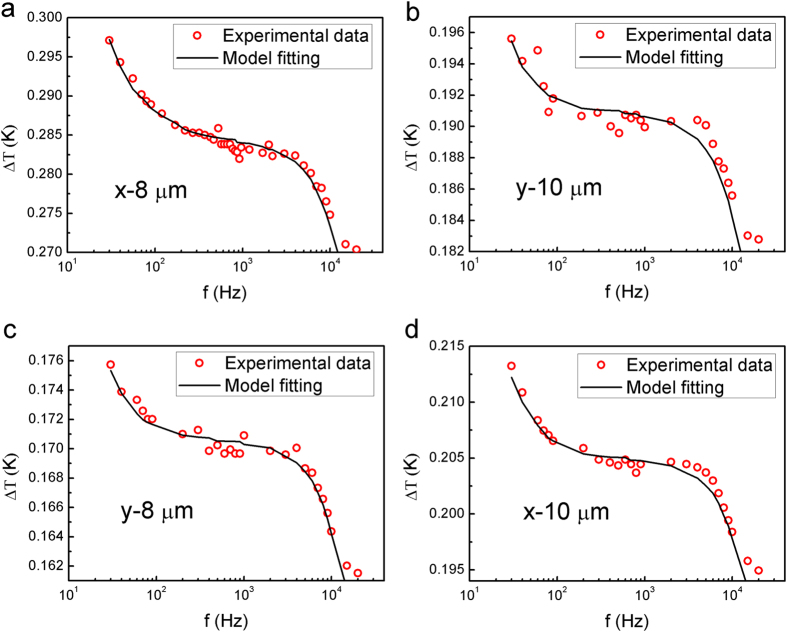
Experimental sensor temperature rise data with model fits. Raw 3 *ω* experimental data along with model fits. Temperature rise data from (**a**) *x*-8, (**b**) *y*-10, (**c**) *y*-8 and (**d**) *x*-10 sensor.

The results from narrow micro-sensors were used to characterize the remaining in-plane thermophysical properties. The penetration depth of the thermal waves meets the requirement of at least 5 times larger than the ratio between the heater half width and the square root of the HACNT array anisotropy^21^ for the *x*-8, *y*-8, *x*-10 and *y*-10 sensor. The experimental real component of temperature rise of the sensors was recorded (Fig. 5) and used to obtain the in-plane thermal conductivity *κ*_*s*2_ and thermal diffusivity *α*_*s*2_ based on the model proposed by Borca-Tasciuc *et al.*^21^. Furthermore, the value of *R*_*c*1_ can be fitted using these temperature rise vs. frequency curves since the corresponding sensitivity coefficients are highest among all analysis results, as shown in Fig. 3b. After *R*_*c*1_ was obtained, the values of *R*_*c*2_ (Fig. 3c) and *n*_*sz*2_ or *α*_*z*2_ (Fig. 3d–f) were least-square fitted using the experimental data for *x*-100 and *y*-100 sensors (Fig. 4), which were remarkably sensitive to these parameters. The measured result for the multi-frequency 3 *ω* data for the HACNT array gives a remarkably high oriented thermal conductivity *κ*_*x*2_ of 127 W/ mK. This is much higher than the highest values reported, ~80 W/mK, for VACNT arrays^3^,^19^. This high thermal conductivity is probably an inherited characteristic from the large thermal transport in 1.2 mm tall VACNTs before shear pressing into HACNTs. Shear pressing enables each column of the CNTs to orderly overlap with their adjacent ones and presents a denser alignment along the *x* direction, as illustrated in Fig. 2f. The thermal conductivity along the *y* axis was surprisingly as high as 42 W/mK, in dramatic contrast to the ~0 thermal conductivity previously reported in VACNT arrays^4^. This is a direct result of spontaneous inter-tube contacts in the form of micro-connections and joints after shear pressing. These contacts help to transfer thermal energy between nanotubes under van der Waals interaction between nanotubes^24^. Similar results were obtained for the thermal diffusivity in three directions, where the highest thermal diffusivity is along the *x* axis: as high as 1.6 × 10^−4 ^m^2^/s, the highest value reported for a CNT array to date. From the experimentally gained data for thermal conductivity and thermal diffusivity, the heat capacity can be easily calculated as 0.8, 0.84 and 0.79 MJ/m^3^K for the *z*, *y* and *x* directions, respectively. Considering the density of the MWCNT is approximately 1.25 g/cm^3^ (see Table 1 for the detailed calculation process), the specific heat is averaged to be 648 J/kgK, close to the reported result for graphite^25^.

**Figure 6:**
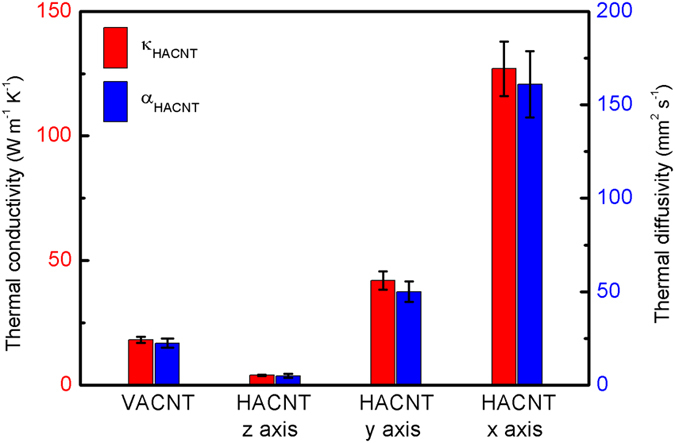
A comparison of the thermal conductivity (*κ*) and thermal diffusivity (*α*) in *z*-direction of the VACNT array and three directions of HACNT arrays. The *x* and *y*-direction thermal conductivity and thermal diffusivity of the VACNT array are close to zero. The error bar is based on the estimated measurement uncertainty for each parameter.

The thermal contact resistance for the HACNT-SiO_2_ interface *R*_*c*1_ was 0.8 ± 0.2 m^2^K/MW, comparable to the best value reported to date for a vertically aligned CNT array with covalent functionalization (0.6 ± 0.2 m^2^K/MW)^26^. The horizontal scheme delicately bypasses the low CNT-SiO_2_ contact area fraction problem resulting from the undesirable differentiated lengths of as-grown VACNTs, and thus facilitates good contact between the CNT array and the SiO_2_ coated heat sink^27^. The thermal contact resistance for the HACNT-Si interface *R*_*c*2_ was measured as 3.5 ± 0.8 m^2^K/MW, close to that reported in the literature for VACNT arrays^4^,^24^,^28^. The measurement uncertainty for thermal characterization was estimated by including the uncertainty of all related quantities (see the detailed calculation process in the Methods Section). Based on propagation of error, the uncertainty of each parameter *X* (*κ*_*z*2_, *κ*_*x*2_, *κ*_*y*2_, *α*_*z*2_, *α*_*x*2_, *α*_*y*2_) was estimated and indicated in Fig. 6.

Since the *xy*-plane thermal transport could be neglected in VACNT array, its thermal measurement just needs the Au sensor with large width (e.g., 100 μm) and is performed via the same procedures and method as that for HACNT film. Based on the equations in Thermal Modelling Section, low-frequency experimental 3 *ω* data was used for directly extracting *z*-direction thermal conductivity and thermal diffusivity for VACNT film. For both VACNT and HACNT thermal measurements, Au sensors were deposited at their top surface, as shown in Fig. 2b for VACNT and Fig. 2e for HACNT. It can be clearly seen that the contact area of HACNT is much larger than that of VACNT. Since the CNT volume fraction in the VACNT array is estimated to be 1.4% (see Table 1) and only approximately 31% of the VACNTs have adequate height to ensure contact with the heat sink (see Figure S2 in Supporting Information), and 0.43% of the total area make good contact with the heat sink. For HACNT, the CNT volume fraction is increased to 10.1%, and nearly all the HACNT at the top surface can closely contact with the heat sink (100%). These results suggest that nearly 10.1% of the total area can make good contact with the heat sink. Therefore, HACNT renders as high as 23 times increase in contact area compared with VACNT, which make the 23 times reduction of thermal contact resistance between CNT array and heat sink, from 18.4 m^2^K/MW for VACNT^4^ to 0.8 m^2^K/MW for HACNT. Moreover, approximately 7 times increase in the volume fraction from VACNT to HACNT means 7 times increase in heat transfer channels, which is the primary responsible for the improved thermal conductivity along CNT height direction.

## Discussion

The remarkably enhanced thermal conductivity along the *x* direction compared with the previous reported values results from swift densification of the CNT thermal transport channels. Numerous experimental data has shown that the apparent thermal conductivity of well-oriented CNT arrays scales up linearly with the increasing number of CNTs (*N*) or the volume fraction (or area fraction for VACNTs, *ϕ*) of CNTs, and a simple estimation equation for describing this relation is^29^,^30^


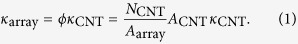


**Figure 7:**
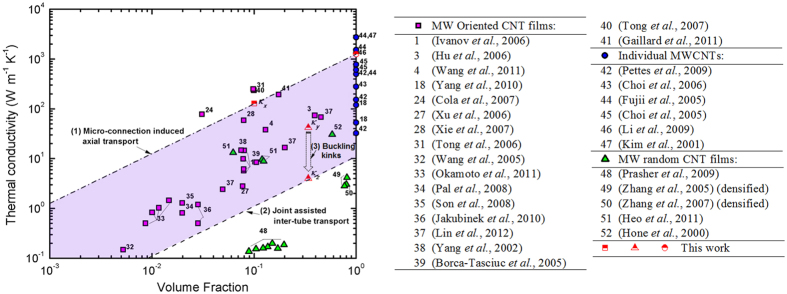
Thermal conductivity as a function of volume fraction for oriented CNT films. These data are determined separately from various thermal characterization techniques^29^. The dash dot line shows the predicted thermal conductivity for an ideal array of CNTs with an individual thermal conductivity of 1257 W/mK, while the dashed line is the predicted value with only inter-tube transport contributing to thermal transport. As the density of HACNT array is considered as a 25× increase compared with the VACNT array, the volume fraction of HACNT array for *y* and *z*-direction is estimated to be ~34% according to the calculation process in Table 1.

The volume fraction for the samples after shear pressing becomes 10.1% as discussed previously. The corresponding spatial density 1.9 × 10^11^ tubes/cm^2^, which is on the same order of the dense good-quality VACNT array by T. Tong *et al.*^31^. (10^10^~10^11^ tubes/cm^2^), shows a desirably high thermal conductivity on the order of 100~200 W/mK for the CNT array. Figure 7 summarizes the majority of the oriented MWCNT samples determined by various thermal characterization techniques^1^,^3^,^4^,^18^,^24^,^27^,^28^,^31^,^32^,^33^,^34^,^35^,^36^,^37^,^38^,^39^,^40^,^41^,^42^,^43^,^44^,^45^,^46^,^47^,^48^,^49^,^50^,^51^,^52^. Our experimental results for the thermal conductivity of HACNT arrays along the principle CNT length direction (*x*) and transverse direction (*y*) present highly desirable thermal dissipation performance, the thermal conductivity values for both directions are close to the upper limits of the reported values for similar CNT assembles.

Understanding the intrinsic nature of HACNT array thermal transport requires a quantitative description based on theoretical calculations, which is critical for further optimization and design of flexible HACNT array-based heat spreading devices. Therefore, quantitative correlations between the nanoscale coupled structures in the HACNT array and their thermal conductivity in three dimensions will also be discussed here. As mentioned above, the *x*-direction thermal conductivity is totally attributed to individual CNT axial transport and the number of effective channels. Based on Eq. (1), the thermal conductivity of an individual MWCNT in our sample is deduced to be as high as 1,257 W/mK, agreeing well with the experimentally measured value for MWCNTs with the same diameter by Li *et al.*^46^. As the thermal conductivity of MWCNTs is dominated by phonon contributions, *κ*~*Cvl*, where *v* is the characteristic sound velocity for the CNT and *l* is the phonon mfp. With *κ* = 1,257 W/mK and *v* = 10^4^ m/s^53^,^54^,^55^,^56^, we have *l* ~ 140 nm, which is smaller than, but still on the same order of, that calculated by Kim *et al.*^47^. This magnitude of mfp which is much larger than the spacing interval between two CNTs indicates the inter-tube overlap has little effect on the axial thermal transport of individual 4-wall CNTs, consistent with molecular dynamics simulation results by Chalopin *et al.* which found the phonon transmission through the CNT was just reduced very slightly when a second CNT crosses the first one^57^. This observation suggests the coupled structure would not reduce the thermal transport ability along the CNT axial direction and thus the thermal transport along the principle CNT length direction is successfully elevated by sevenfold owing to the increased packing density after shear pressing.

**Figure 8:**
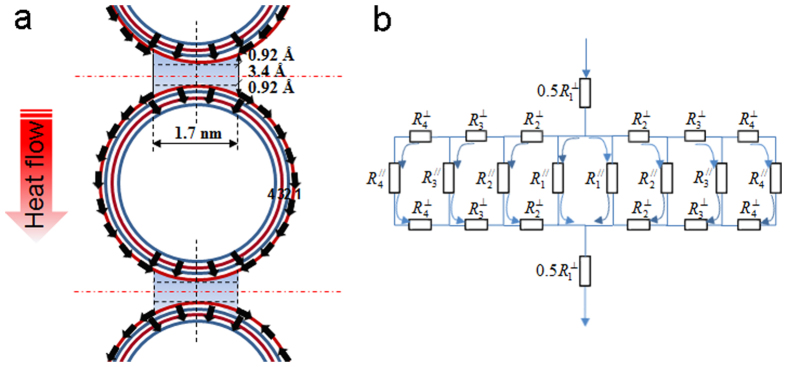
(**a**) Schematic representation of superimposed 4-wall CNTs with van der Waals interaction helping heat transfer between CNTs. (**b**) Intra-shell and inter-shell thermal resistance network as an electrically equivalent circuit of resistors. Resistors 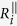 along the graphene layer represent the thermal resistance of shell sections participating in heat transport with length *l* and cross section 0.34 × *l* nm^2^. Resistors 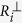 connected perpendicular to 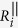 represent the thermal resistance between shells in the radial direction. Arrows show the heat current directions.

Furthermore, our structure coupling functionalization is also favorable to thermal transport along the *y* and *z* directions. Two typical coupling structures: micro-connections and joints, provide additional transport paths besides inter-tube transport. The role of micro-connections is considered to provide the possibility of CNT axial transport along the *y* and *z* directions, which greatly improves thermal transport ability and thus can be estimated as the upper limit for thermal conductivity along that direction (see Fig. 7). The joints help to shorten the gap between CNTs and enable inter-tube heat transport. The inter-tube heat transport path is schematically modelled in Fig. 8a. Unlike sequential transport between graphene layers for graphite, generally at a low phonon transport velocity, the round shape of graphene in MWCNTs enables phonon transport along graphene layers at a much higher velocity. The calculation process for the sizes of the inter-tube interacting structure are introduced in the Methods Section in detail. The corresponding total thermal resistance network along the *z* direction is schematically described in Fig. 8b. The heat carrier transport within a single MWCNT is typically via two routes, i.e., tunneling (⊥) and circum-navigating (//) processes. Phonon vibrations can tunnel from one shell to another, similar to the out-of-plane thermal transport in graphite. We use the cross-plane thermal conductivity for graphite at room temperature, 5.7 W/mK^30^, for the calculation of the thermal resistance in the tunneling process. As all phonon transport paths for circum-navigating processes are shorter than the mfp of MWCNT, the thermal transport could be ballistic. Therefore, the thermal conductivity 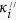 for this transport varies for different shells owing to size effects, and these values could refer to the results in our previous studies^11^ and others’^53^,^54^,^55^,^56^ without taking into account defect scattering. The total effective thermal resistance based on the network is calculated as 0.0915/*l* using the parameters listed in Table 2, and the transport unit defined as a 4-wall CNT and the interaction zones on both sides (region circled by the red dashed lines in Fig. 8a) has a total effective thermal resistance of 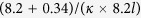. Therefore, the upper limit for thermal conductivity along the *z* direction is estimated as 11 W/mK. This value is considered as the lower limit for thermal conductivity along the *y* direction as indicated in Fig. 7. It can be clearly seen that the obtained *y*-direction thermal conductivity *κ*_*y*_ is a resultant of two transport contributions: (1) micro-connection induced axial transport and (2) joint assisted inter-tube transport.

The *z*-direction thermal transport mechanism is similar to that of the *y*-direction except bending buckling kinks at the bottom induce additional thermal resistance. These extra “thermal resistors” dramatically reduce the thermal conductivity to 4.0 W/mK, nearly a tenfold reduction compared with the *y*-direction thermal conductivity, which is close to predication results based on computational simulation^19^,^20^. Although the *z*-direction thermal conductivity displays a fourfold reduction compared to the as-grown VACNT counterpart before shear pressing, the total thermal resistance consisting of the HACNT thermal resistance, the thermal contact resistance at the HACNT-SiO_2_ and HACNT-Si interfaces is much smaller than the VACNT counterpart. Therefore, the *z*-direction thermal transport for our functionalized flexible HACNT array-based film is also enhanced.

In summary, our result suggests the mechanical scheme “shear pressing” can maintain ordered alignment of CNTs unlike the disordered structure in buckypaper or in a CNT mat. The structure-coupling nature of CNTs has a decisive effect on thermal transport in nanoscale systems. We have shown that transforming the VACNT array into a HACNT substantially increases the in-plane thermal transport and reduces the overall thermal contact resistances for the HACNT array and its interfaces thermal contact resistance. Our functionalization scheme for CNT arrays is simple and stable against aging. This development makes CNT film promising to function as anisotropic heat spreader with great advantages over conventional metal counterpart especially in eliminating hot spots. Therefore, the variant of VACNT, i.e. HACNT proposed in this study will potentially help with the optimization of heat dissipation devices for realizing efficient microelectronic systems.

## Methods

### CNT growth

MWCNT arrays are grown directly on 10 mm × 10 mm surface-polished (100) Si wafer substrates with a thickness of 344 μm by CVD using both a quartz tube furnace and a nano-particle catalyzed synthesis system. A detailed description of the fabrication process can be found elsewhere^6^,^9^. Briefly, a 10/2 nm thick Al_2_O_3_/Fe buffer layer/catalyst film was deposited onto the Si wafer by magnetron sputtering. Vertically aligned MWCNT arrays were grown at 750 °C using ethylene as the carbon source. Based on the statistical analysis of TEM images, the CNTs typically have 2–4 walls with outside diameters of approximately 8.2 nm and lengths of ~1.2 mm^6^,^9^.

### VACNT alignment parameters

The VACNT array alignment presents several useful parameters for the subsequent estimation of volume fraction of CNT packing in HACNT array. Therefore, we need to determine all these parameters. Statistical analysis based on TEM images gave the average values for four parameters for as-grown VACNTs: inner/outer diameter, number of walls and wall thickness^6^. CNT array density was estimated based on the measured macroscopic dimensions of the array and the mass of the array. To obtain the mass of the array, the mass of the substrate was measured prior to VACNT growth and subtracted from the total mass after growth^6^. All the mass measurements were performed using a sensitive microgram balance. Our VACNT samples for shear pressing are estimated to have a volume/area fraction of CNTs of 1.4% through the comparison of the density of a CNT array^6^ to the density of an individual CNT. Table 1 summarizes all the parameters and estimated results.

The distance between two adjacent CNTs and the cut-off distance for the van der Waals interactions^17^,^18^ can be quantitatively estimated. The distance between two parallel identical CNTs at equilibrium state is 3.4 Å, and the van der Waals interactions almost vanishes at 4–5 Å^17^. The Lennard-Jones potential continuum model^17^ has been proven effective for estimating the binding energy for graphitic structures. It is used here to estimate the effective interacting segment of nanotube circumference. By performing numerical integration of the surface area under an attractive potential curve above the equilibrium distance, the normalized tube separation is estimated to be 1.54, corresponding to (*R*-*r*) = 5.236 Å. For a MWCNT with an outermost shell diameter of 8.2 nm, the effective separation Δ*x*_eff_ = (*R*-*r*) −3.4 Å = 1.84 Å. Hence, the increase of separation from equilibrium to Δ*x*/2 = 0.92 Å corresponds to the effective interaction segment of 1.7 nm, shown in Fig. 8a.

### Functionalization and structural details

For functionalization, the as-grown VACNT arrays on Si substrate were mounted on a pressing device with adjustable inclined angles for shear pressing. A detailed description of the device can be found elsewhere^9^. Briefly, a parallel aluminum plate pressed VACNT arrays into HACNT arrays at an angle of 35° within an operation time of about a couple of seconds. The CNTs’ vertical orientation was uniformly transformed into horizontal orientation while preserving the alignment due to the high friction interface between the CNT array and the aluminum plate. A large amount of empty space within the CNT array was eliminated after the process because CNTs would spontaneously lay on each other at a small off-axis angle to the plane of the flat substrate. Those horizontally arranged long CNTs also partly touch adjacent ones when towed to different planes or tangled to form joints. All these structure coupling behaviors actually help to provide additional bypass or channels for thermal transport. Surface functionalization of the HACNT arrays was realized by simultaneous replacement of CNT tips using their cylindrical surface and carefully polishing the CNT array top before depositing onto the SiO_2_ film to insure intimate contact between the CNT array and SiO_2_ film.

### Thermal characterization

We used a modified version of a 3 *ω* method^21^,^23^,^38^ to characterize anisotropic thermal transport and thermal contact resistance. The 3 *ω* method requires deposition of micro sensors on the sample surface to be investigated before measurement. For the deposition of the 200 nm thick Au sensors, in order to prevent metal particles from going into the inner part of the measured sample, we firstly fabricated a 10/95 nm thick Ti/SiO_2_ layer via magnetron sputtering on the top of measured samples, i.e., HACNT and VACNT, and then sputtered the Au film and patterned it into a certain layout using a photolithography technique on the surface of SiO_2_ layer. Although the 3 *ω* technique used here evaluates the sum of thermal resistances of all layers involved, including SiO_2_ layer (0.07 m^2^K/MW), CNT film (67 m^2^K/MW for VACNT, 11 m^2^K/MW for HACNT), SiO_2_-CNT interface (0.8 ± 0.2 m^2^K/MW) and CNT-Si interface (3.5 ± 0.8 m^2^K/MW), the share occupied by SiO_2_ layer is much smaller compared with CNT film. Therefore, the influence of SiO_2_ layer could be neglected and would not degrade the thermal measurement for CNT film. In addition, the deposition of SiO_2_ layer on the top of the CNT film as well as the following fabrication of Au sensors on the surface of SiO_2_ layer based on photolithography technique are very mature and can ensure good contacts between Au-SiO_2_-CNT multilayer structures. The reasonable and stable electrical resistance of Au sensors observed during the measurements also indicated the test structures are suitable and appropriate.

The micro-sensor group consists of four sensors laid out in a particular pattern on the top of two HACNT array samples (see Figure S3 in Supporting Information). For sample 1 with thickness of 40 μm, a 40 μm wide sensor and a 8 μm wide sensor were placed along the direction parallel to the CNT’s axis (*x* direction), while a 100 μm wide sensor and a 10 μm wide sensor were placed along the direction perpendicular to the CNT’s axis (*y* direction). For sample 2, with thickness of 45 μm, the placements of the four sensors were exchanged. We used two placements to simplify the method for extracting anisotropic thermal conductivity.

The test configuration was placed in a temperature-controlled chamber and connected to the electrical circuit for the 3 *ω* method as shown in our previous literature^11^. The Au micro-sensor serves both as a heater and a thermometer. Driven by the sinusoidal voltage out of a signal generator at an angular frequency  *ω*, the Au strip generates Joule heat and produces a temperature oscillation at 2 *ω*, further resulting in a modulated electrical resistance at 2 *ω*. Combined with the driving current at  *ω*, the voltage response along the Au strip consists of a modulated component at 3 *ω*. Based on this principle, the experimental temperature rise of the Au strip can be calculated using the 3 *ω* and the 1 *ω* components of voltage^58^ directly obtained from a commercial lock-in amplifier and temperature coefficient of resistance (TCR) of Au, *α*_*CR*_.


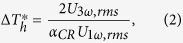


Another type of parameter relating the experimental signal to the theoretical quantity is thermal impedance:^22^


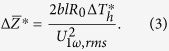


The experimental setup was first calibrated using a standard sapphire sample whose properties had been well characterized. The deduced anisotropic thermal conductivity in three dimensions agrees well with manufacture provided values^59^. The values of the TCR for the Au sensors were verified by recording their electrical resistances versus temperature curves in a temperature controllable chamber. The TCRs for the Au sensors were all around 0.004 K^−1^.

### Thermal modeling

Low-frequency (0.1~3 kHz) experimental 3 *ω* data using *x*-100, *y*-100, *x*-40 and *y*-40 sensors was compared with model calculations based on the two-dimensional film-on-substrate thermal impedance model of Tong *et al.*^23^. This approach presumes the impedance change induced by the HACNT array layer is equivalent to a combination of the modified bulk film resistance and the reciprocal of the interface thermal conductance (1/*h*, namely *R*_*c*_), which is applicable to our sensors with width larger than the HACNT array thickness. The average of Δ*Z* over the entire Fourier space is expressed as


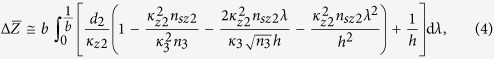


The above equation can be further arranged as


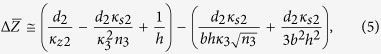


As *κ*_3_ is much larger than *κ*_*z*2_ for our test structure, Eq. (5) can be approximated as


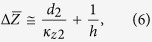


The thermal impedance for the four test structures can thus be rearranged as the following matrices operation,


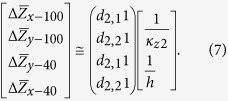


where *d*_2,1_ = 40 μm, *d*_2,2_ = 45 μm. The matrix formulation is especially powerful in simultaneously extracting the two unknown parameters. The subscript of 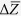 denotes the direction along which the sensor is placed and the width of the sensor. Therefore, *κ*_*z*2_ and *R*_*c*1_ were obtained through the two cross measurements using *x*-100, *y*-100, *x*-40 and *y*-40 sensors. And the two groups of comparative measurement double check the results.

High-frequency (3~10 kHz) experimental 3 *ω* data using *x*-10, *y*-10, *x*-8 and *y*-8 sensors was compared with model calculations based on the one-dimensional line-source-on-anisotropic substrate heat conduction model of Borca-Tasciuc *et al.*^21^. This model based on the assumption that the thermal penetration depth meets the requirement of at least 5 times larger than the ratio between the heater half width and the square root of HACNT array anisotropy 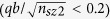. Two in-plane parameters *κ*_*s*2_ and *α*_*s*2_ can be obtained based on the experimental real component of the temperature rise of the sensors:


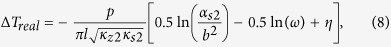


*R*_*c*1_ was fit using these temperature rise vs. frequency curves over the full experimental frequency range since the corresponding sensitivity coefficients are highest among all analysis results. Experimental 3 *ω* data using the *x*-100 and *y*-100 sensors was compared with model predictions based on the two-dimensional multilayer heat conduction model of Borca-Tasciuc *et al.*^21^ combined with analysis including the interlayer TBR of Olson *et al.*^22^. A least-square minimization procedure was used to adjust the two remaining parameters *R*_*c*2_ and *n*_*sz*1_ or *α*_*z*2_ to fit the experimental data. The combined model is as follows:


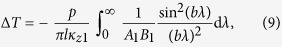


where


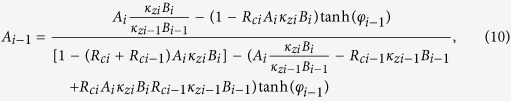



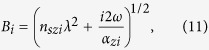



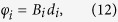



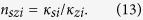


In the above expressions, *s* may denote *x* or *y* depending on the sensor layout, subscript 1, 2, 3 denotes the layer from top to bottom, that is SiO_2_, HACNT array and silicon, respectively, *b* is the strip half width, *λ* is the Fourier transform variable in the *s* direction denoting the spatial frequency, *κ* is the thermal conductivity, *p*/*l* is the peak electrical power per unit length,  *ω* is the angular frequency of the alternating current, *d* is the thickness, *n*_*sz*_ is the ratio of the in-plane to cross-plane thermal conductivity, and *α* is the thermal diffusivity.

### Measurement uncertainty estimation

According to the analysis by Hu *et al.*^3^ the measurement uncertainty for the present 3 *ω* method is mainly attributed to the uncertainty of the third harmonic voltage *U*_3_ *ω*_,*rms*_ (estimated as 2.1% based on the standard deviation) and the TCR for the Au sensors (calculated as 1.2%). *U*_1_ *ω*_,*rms*_ is found to be very accurate (<0.1%). Based on Eq. (2), the uncertainty of the experimental temperature rise *δ*ΔT^*^/ΔT^*^ was calculated to be 2.4%. Therefore, the uncertainty of any parameter to be extracted *X* (*κ*_*z*2_, *κ*_*x*2_, *κ*_*y*2_, *α*_*z*2_, *α*_*x*2_, *α*_*y*2_, *R*_*c*1_, *R*_*c*2_) can be estimated according to the principle of uncertainty propagation,


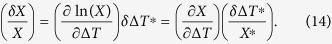


The quantity in the first parentheses on the right side of the above equation can be estimated by calculating the deviation of data fitting after applying a small offset of the change in temperature rise.

## Additional Information

**How to cite this article**: Qiu, L. *et al.* Remarkably enhanced thermal transport based on a flexible horizontally-aligned carbon nanotube array film. *Sci. Rep.* **6**, 21014; doi: 10.1038/srep21014 (2016).

## Supplementary Material

Supplementary Information

## Figures and Tables

**Table 1:** Experimentally obtained parameter and estimated volume fraction for VACNT array.

Experimentally obtained parameter		Calculated results	
Inner diameter	5.5 nm	Cross-section area of hollow part	π(2.75)^2^ = 23.8 nm^2^
Number of walls	4	Cross-section area of whole CNT	π(4.1)^2^ = 53.1 nm^2^
Wall thickness	0.34 nm	Wall area	(53.1–23.8) = 29.3 nm^2^
Outer diameter	8.2 nm	Volume fraction of wall area in tube	*area*_wall_/*area*_total_ = 0.552
Array density	0.017 g/cm^3^	Density of as-grown CNT	2.26 g/cm^3^ × 0.552 = 1.25 g/cm^3^
graphite density	2.26 g/cm^3^	Volume fraction of array	(0.017 g/cm^3^/1.25 g/cm^3^)*100 = 1.4%

**Table 2:** Geometrical parameter used for simulation of thermal resistance network for 4-wall CNT array.

#	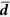 (nm)	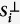 (nm^2^)	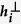 (nm)	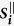 (nm^2^)	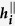 (nm)	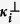 (W/m K)	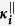 (W/m K)	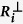 (K/W)	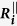 (K/W)
1	(8.2 + 7.5)/2 = 7.9	1.71*l*	0.34	0.34*l*	10.7	5.7	114^*^	0.0348/*l*	0.276/*l*
2	(7.5 + 6.8)/2 = 7.2	1.72*l*	0.34	0.34*l*	9.6	5.7	103^*^	0.0348/*l*	0.274/*l*
3	(6.8 + 6.2)/2 = 6.5	1.72*l*	0.34	0.34*l*	8.5	5.7	91^*^	0.0347/*l*	0.275/*l*
4	(6.2 + 5.5)/2 = 5.9	1.72*l*	0.34	0.34*l*	7.5	5.7	80^*^	0.0346/*l*	0.276/*l*

^*^Note: these values are estimated based on tensor equation of *κ* for crystalline solid with 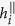 as the separation of two scattering boundaries without taking into defect scattering account^11^,^53^,^54^,^55^,^56^.
