# Supplementary material for: Remarkably enhanced thermal transport based on a flexible horizontally-aligned carbon nanotube array film
Source: Sci Rep. 2016 Feb 16;6:21014. doi: 10.1038/srep21014 (PMC4754700; doi:10.1038/srep21014)
Supplement: Supplementary Information [file srep21014-s1.doc]

**Supporting Information:**

Remarkably enhanced thermal transport based on a flexible horizontally-aligned carbon nanotube array film

*Lin Qiu,^†,‡,§^ Xiaotian Wang,^˥,§^ Guoping Su,^†,¦^ Dawei Tang,^*,†^ Xinghua Zheng,^*,†,||^ Jie Zhu,^†^ Zhiguo Wang,^┴^ Pamela M. Norris,^‡^ Philip D. Bradford,^¶^ and Yuntian Zhu^¶,#^*

^†^Institute of Engineering Thermophysics, Chinese Academy of Sciences, Beijing, 100190, People’s Republic of China

^‡^Department of Mechanical and Aerospace Engineering, University of Virginia, Charlottesville, VA 22904-4746, USA

^˥^School of Materials Science and Engineering, Nanyang Technological University, 50 Nanyang Avenue, Singapore 639798, Singapore

^¦^Shenhua Guohua (Beijing) Electric Power Research Institute Co., Ltd., Beijing 100025, People’s Republic of China

^||^Department of Mechanical Engineering, University of California, Riverside, CA 92521, USA

^┴^China National Electric Engineering Co., Ltd., Beijing 100048, People’s Republic of China

^¶^Department of Materials Science and Engineering, North Carolina State University, Raleigh, NC 27695, USA

^#^School of Materials Science and Engineering, Nanjing University of Science and Technology, Nanjing 210094, People’s Republic of China

----------------------------------------------------

*Corresponding author: Prof. Dawei Tang, Tel: +86-10-82543020, Fax: +86-10-82543022, Email: [dwtang@mail.etp.ac.cn](mailto:dwtang@mail.etp.ac.cn); Associate Prof. Xinghua Zheng, Tel: +86-10-82543022, Fax, +86-10-82543022, Email: jzzhengxinghua@163.com.

**Contents**

1. HACNT on Si wafer structure.
2. Tip height distribution of as-grown VACNTs.
3. Photographic image of 3*ω* heater/sensor group for thermal characterization.
4. HACNT on Si wafer structure.

A low-resolution SEM image clearly shows the whole image of HACNT on Si wafer structure and the thickness of HACNT film is approximately 45 μm.


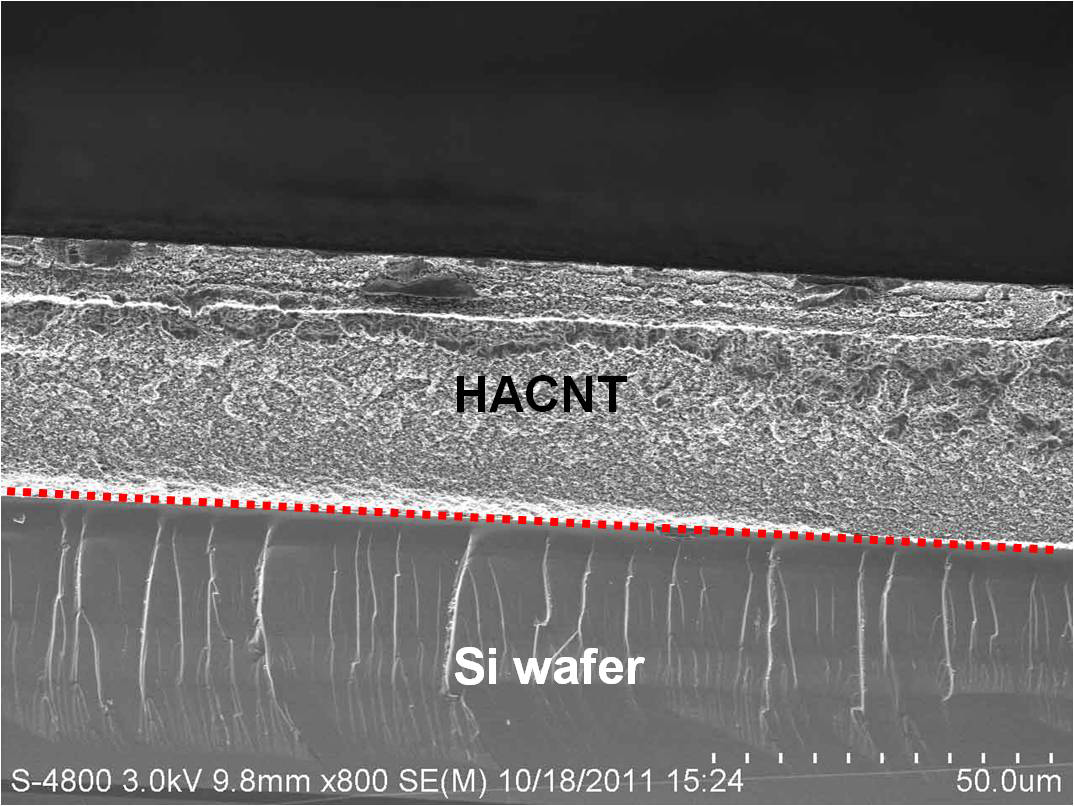


**Figure S1. Low-resolution SEM for showing the whole thickness of HACNT on Si wafer structure.**

1. Tip height distribution of as-grown VACNTs.

Figure S2 shows the tip height distribution of the as-grown VACNT array within the top 940 nm region. It is clearly indicated that a large height difference exists at the array tip, from ~150 nm to 940 nm. This broad height distribution is the main reason for large thermal contact resistance between VACNT and the heat sink. In contrast, much more flattened surface for HACNT array (Figure 2e) will contribute to significant reduction of the thermal contact resistance.


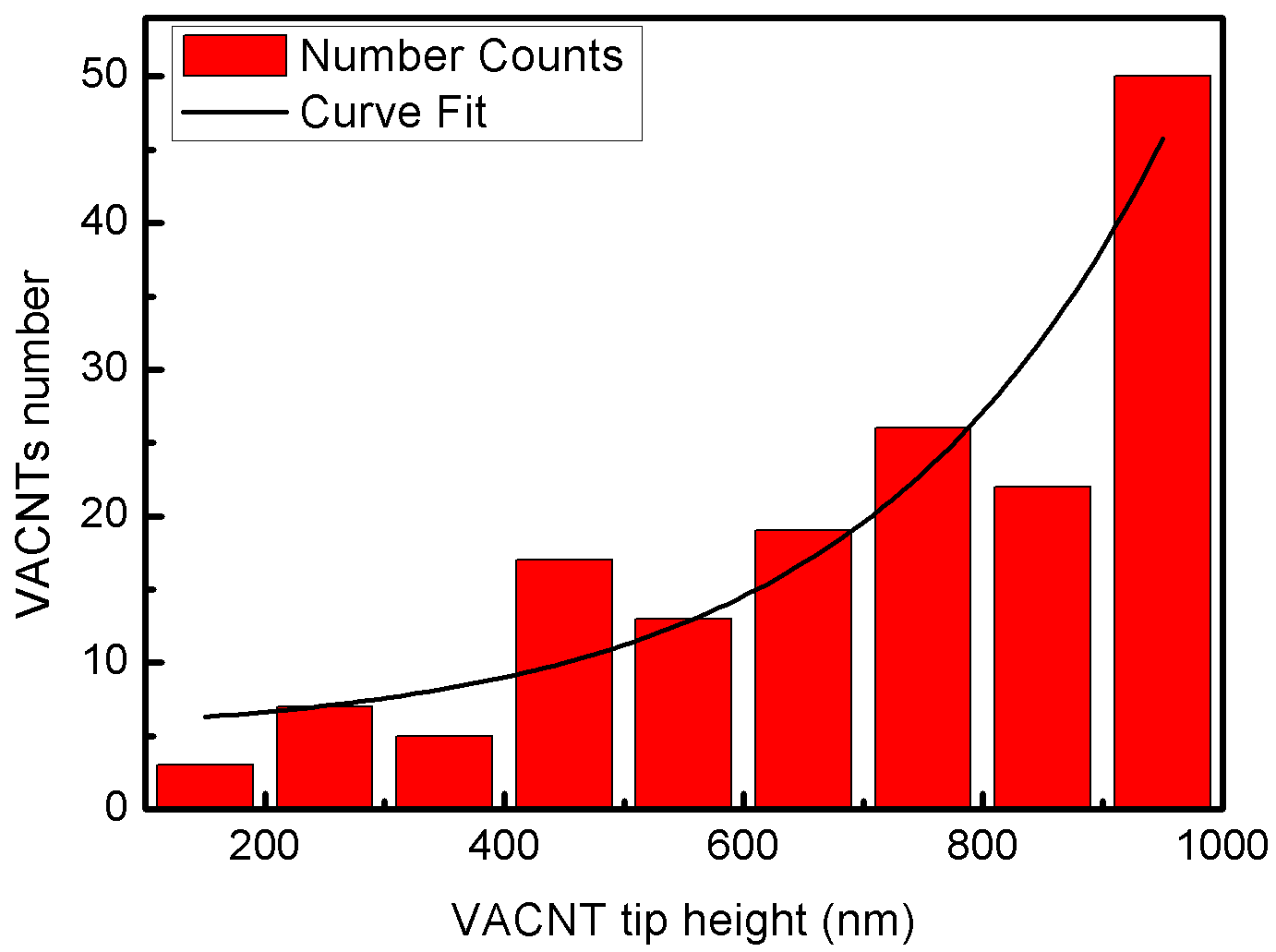


**Figure S2. Tip height distribution of as-grown VACNT. The statistics analysis is within a ~ 940 nm height region.**

1. Photographic image of 3*ω* heater/sensor group for thermal characterization.

A 10/95 nm thick Ti/SiO_2_ adhesion/insulation film was firstly made on the top of the HACNT arrays using magnetron sputtering. Then a 200 nm thick Au film was sputtered and patterned into a certain layout using a photolithography technique.


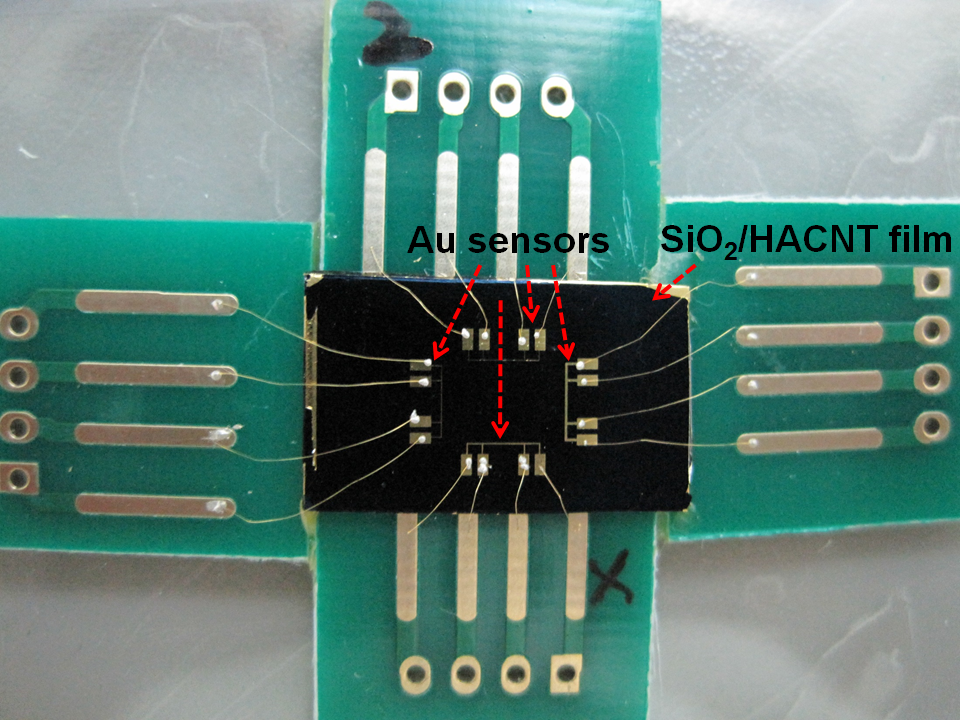


**Figure S3. Photographic image of the 3ω heater/sensor group deposited on SiO_2_/HACNT film.**
